# MicroRNA-7 inhibition rescues age-associated loss of epidermal growth factor receptor and hyaluronan-dependent differentiation in fibroblasts

**DOI:** 10.1111/acel.12167

**Published:** 2013-11-12

**Authors:** Adam C Midgley, Timothy Bowen, Aled O Phillips, Robert Steadman

**Affiliations:** Institute of Nephrology, Institute of Molecular & Experimental Medicine, School of Medicine and Cardiff Institute of Tissue Engineering & Repair, University of CardiffHeath Park, Cardiff, CF14 4XN, UK

**Keywords:** epidermal growth factor receptor, fibroblast, hyaluronan, microRNA-7, transforming growth factor-β1, wound-healing

## Abstract

Age-related defects in fibroblast differentiation were previously shown to be associated with impaired hyaluronan synthase 2 (HAS2) and epidermal growth factor receptor (EGFR) function, with both required for normal fibroblast functionality. In fibroblasts, transforming growth factor-beta 1 (TGF-β1)-dependent phenotypic activation uses two distinct but co-operating pathways that involve TGF-β receptor (TGF-βR)/Smad2 activation and HA-mediated CD44-EGFR co-localization and signalling through extracellular signal-regulated kinase 1/2 (ERK1/2). The HA-mediated CD44-EGFR pathway was found to be compromised with *in vitro* aging, through loss of EGFR expression and a reduced movement of CD44 throughout the cellular membrane. Here, we also investigate the involvement of microRNAs (miRNAs) in age-related loss of differentiation, through investigation of miRNA-7 (miR-7) regulation of the HA-mediated EGFR-signalling pathway. The transcription of miR-7 was found to be upregulated in aged cells. In young cells, age-related loss of differentiation could be mimicked through transfection of pre-miR-7, and in aged cells, could be reversed through transfection of locked nucleic acids (LNA) targeting miR-7. Additionally, miR-7 was found to be involved in the regulation of CD44 membrane motility, which was downregulated in instances of miR-7 upregulation, and partially restorable through either miR-7 inhibition or HAS2 overexpression. The altered dynamics of CD44 in the cell membrane demonstrated a further action of miR-7 in regulating the HA-dependent CD44/EGFR pathway. We explain this novel mechanism of age-associated functional consequence due to miR-7 upregulation and demonstrate that it is reversible; highlighting miR-7 as a potential target for restoring the healing capabilities in chronic wounds in the elderly.

## Introduction

Wound healing, regardless of the aetiology of the wound, involves an overlapping sequence of events that culminate in remodelling of the extracellular matrix and restoration of tissue integrity. Fibroblasts are central to this and when activated by transforming growth factor-β1 (TGF-β1), they undergo phenotypic transition into α-smooth muscle actin (α-SMA) positive, contractile myofibroblasts (Gabbiani, 2003). These myofibroblasts are responsible for wound closure and the formation of the collagen-rich scar. It has been demonstrated previously that fibroblasts isolated from aging skin have impaired migration, impaired proliferative response and defects in matrix generation compared to those from young skin (Ashcroft *et al*., 1995; Shiraha *et al*., 2000). Furthermore, we have shown that aging cells are resistant to TGF-β1-triggered fibroblast to myofibroblast differentiation, despite the normal activation of the TGF-β1 intracellular signalling pathways (Simpson *et al*., 2009, 2010).

We have previously demonstrated that hyaluronan (HA), synthesized by HA synthase (HAS) 2, is a key factor in the regulation of fibroblast differentiation (Meran *et al*., 2007; Webber *et al*., 2009). In addition, HA is involved in mediating cellular responses to TGF-β1. For example, our previous studies in epithelial and fibroblast cells have demonstrated that HA modulates TGF-β1 signalling after interaction with its receptor, CD44, promoting CD44-epidermal growth factor receptor (EGFR) co-localization and downstream signalling (Ito *et al*., 2004a,b; Simpson *et al*., 2010). Age-related failure of TGF-β1- induced differentiation to the myofibroblast phenotype is associated with the inability to induce HAS2, a decrease in HA synthesis, and a lack of pericellular coat formation. HA synthesis and coat assembly could be restored by forced expression of HAS2 followed by TGF-β1 stimulation (Simpson *et al*., 2009). This did not, however, restore the myofibroblast phenotype, and studies, including our own, have demonstrated that the loss of EGFR expression and its responsiveness in aged cells is central to age-related resistance to differentiation (Shiraha *et al*., 2000; Simpson *et al*., 2010).

MicroRNAs (miRNAs) are small, single-stranded and looped 18–23 nucleotide RNA molecules that function as regulators of gene expression by binding and actively inhibiting the expression of mRNA to moderate cell function such as proliferation and differentiation. The microRNA, miR-7 has been shown to antagonize EGFR expression (Kefas *et al*., 2008), to be involved in EGFR-related cancer progression (Webster *et al*., 2009; Chou *et al*., 2010), and to regulate EGFR-mediated development (Li & Carthew, 2005). However, whether or not miR-7 is implicated in age-associated loss of EGFR and therefore fibroblast resistance to differentiation has yet to be investigated. Our hypothesis is that age-related miR-7 upregulation may be central to the loss of the HA-dependent EGFR signalling pathway. Herein, we show that miR-7 affects the cellular expression and functionality of EGFR implicating a role in the cellular resistance to differentiation and detail the functional consequences arising from miR-7 upregulation in young cells. In addition, we demonstrate that inhibition of miR-7 can rescue the differentiation response in *in vitro* aged fibroblasts. Furthermore, we describe a novel mechanism in which miR-7 regulates the HA-mediated CD44/EGFR signalling pathway through loss of CD44 cellular membrane motility. We also show that miR-7 inhibition can restore CD44 movement in a similar manner to HAS2 overexpression, thus highlighting how miR-7 can indirectly regulate HA and CD44 and the subsequent loss of differentiation in response to TGF-β1 stimulation.

## Results

### Aging fibroblasts have decreased expression of EGFR mRNA and protein but maintain promoter activity

**Figure 1:**
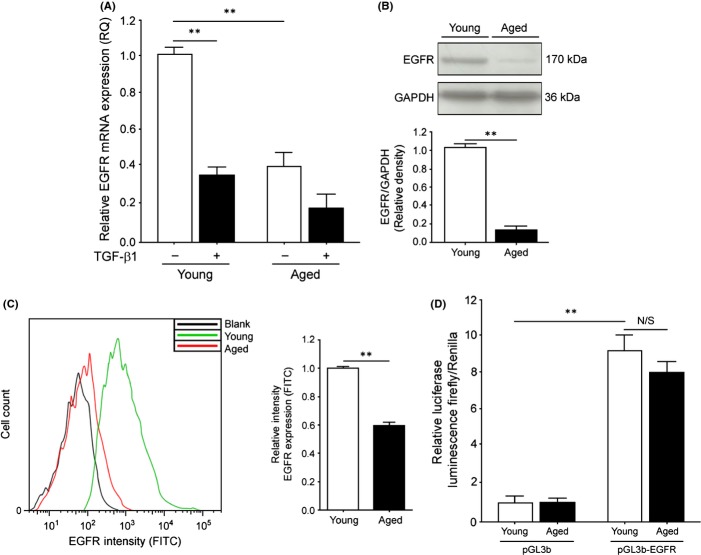
EGFR expression is downregulated in aging of fibroblasts. (A) Young and aged fibroblasts were grown to confluent monolayers and were growth arrested for 48 h. Cells were then incubated in serum-free medium alone or in medium containing 10 ng/mL TGF-β1 for 72 h. The expression of EGFR mRNA was examined by QPCR and results are shown as the mean ± SEM of three individual experiments. (B) Western blot analysis of total EGFR protein in young and aged fibroblasts. GAPDH was used as a loading control. Representative blot is shown and densitometry graph shown is the mean ± SEM of three individual experiments. (C) Flow cytometry analysis of cell surface expression of EGFR in young (green) and aged (red) fibroblasts. Unlabelled cells (black) were used as intensity controls. Bar graph of relative intensity shown is the mean ± SEM of three individual experiments. (D) Young or aged fibroblasts were grown to 70% confluence and transiently transfected with either an empty pGL3b vector or pGL3b-EGFR. Luminescence was measured averaged. Cells were co-transfected with Renilla to measure efficiency and to normalize data. Graph shown is the mean ± SEM of three individual experiments. N/S = no significance, **P ≤ 0.01. EGFR, epidermal growth factor receptor.

Previous studies, including our own (Shiraha *et al*., 2000; Simpson *et al*., 2009, 2010) have demonstrated a loss of EGFR that correlates with increasing fibroblast population doubling. However, it is not clear what mechanism dictates this loss of expression. To determine the extent of EGFR loss in our cell system, we analysed EGFR mRNA, total protein, cell surface expression and constitutive EGFR promoter activity in young and aged fibroblasts. EGFR mRNA analysed by quantitative PCR (QPCR) was found to be significantly down-regulated in aged fibroblasts (Fig. 1A). This was reflected in age-associated loss of both EGFR total protein, as determined by Western blot (Fig. 1B), and cell surface expression, as determined by flow cytometry (Fig. 1C). Interestingly, when the activity of the EGFR promoter using a luciferase reporter construct was tested, no observable differences in luminescent intensity were observed between young and aged cells (Fig. 1D), suggesting that the downregulation of EGFR mRNA and protein were not necessarily a result of reduced levels of transcription factors binding to the EGFR promoter, and thus, the aged-associated loss of EGFR could be a consequence of post-transcriptional activity.

### Cellular membrane mobility of CD44 is lost in aged fibroblasts

**Figure 2:**
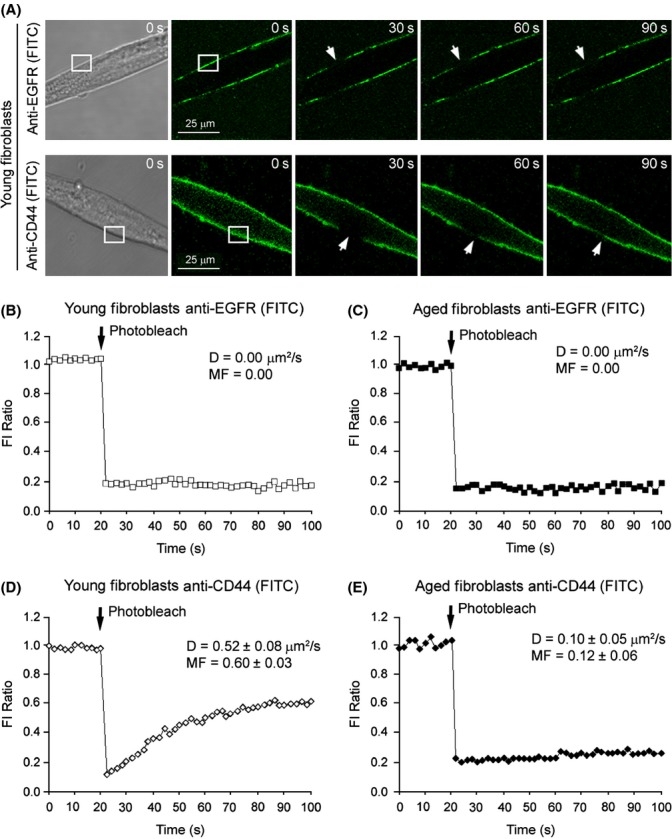
CD44 cellular membrane mobility is reduced in aging fibroblasts. (A) Sample time-lapse series of fluorescent recovery after photobleaching (FRAP) experiments. Original magnification ×630. Young or aged fibroblasts were grown to 70% confluent monolayers on 22 mm diameter glass coverslips in 35 mm six-well tissue culture plates. Cells were growth arrested in serum-free medium for 48 h. FRAP was performed at 37 °C by photobleaching an approximately 10 μm area of the cell membrane (indicated by white boxes). The recovery of fluorescence into this area (indicated by white arrows) was quantified and expressed as a fraction of the fluorescence intensity (FI) of a second region of membrane outside of the photobleached area (FI Ratio). Complete quantified time-courses, average diffusion constants (D) and mobile fractions (MF) are shown for: (B) EGFR in young fibroblasts; (C) EGFR in aged fibroblasts; (D) CD44 in young fibroblasts and E. CD44 in aged fibroblasts. All results shown are mean ± SEM of five independent experiments. EGFR, epidermal growth factor receptor.

The loss of EGFR expression in aged fibroblasts has been reported to impact on a reduced differentiation potential through a loss of the interaction between CD44 and EGFR, an important step in driving fibroblast to myofibroblast transformation (Simpson *et al*., 2010; Meran *et al*., 2011; Midgley *et al*., 2013). We previously highlighted the importance of CD44 membrane motility in contribution to EGFR interaction (Midgley *et al*., 2013). Here, we examined whether the receptor behaviour of EGFR altered in aged cells, and whether the ability of CD44 to move within the cell membrane was also affected using fluorescence recovery after photobleaching (FRAP). Representative FRAP images are shown for young fibroblast EGFR and CD44 (Fig. 2A). EGFR was found to remain within static domains across the membrane regardless of cellular age (Fig. 2B,C). In contrast, while CD44 had the potential to move throughout the membrane in young fibroblasts (Fig. 2D), this was lost in aged fibroblasts (Fig. 2E). These data suggest that CD44 mobility could be another contributor to the age-associated loss of differentiation and could explain why previously overexpressed EGFR alone (Simpson *et al*., 2010) was not sufficient to fully restore aged fibroblast differentiation to myofibroblasts.

### MicroRNA-7 targets the 3′UTR of EGFR and is upregulated in aged fibroblasts

**Figure 3:**
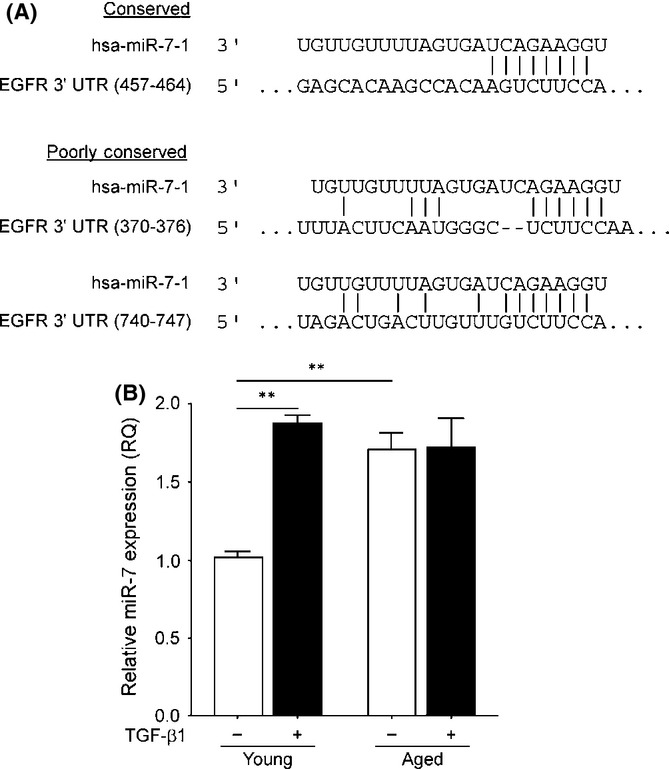
MicroRNA-7 targets 3′UTR of EGFR mRNA and is upregulated in aging fibroblasts. (A) Highly conserved and poorly conserved microRNA-7 (miR-7) seed sites on the 3′UTR of EGFR mRNA as determined by *in silico* analysis with TargetScan v6.2 (Whitehead Institute, Cambridge, MA, USA). (B) Young and aged fibroblasts were grown to confluent monolayers and were growth arrested for 48 h. Cells were then incubated in serum-free medium alone or in medium containing 10 ng/mL TGF-β1 for 72 h. The expression of miR-7 was examined by QPCR, and results are shown as mean ± SEM of three individual experiments. **P ≤ 0.01. EGFR, epidermal growth factor receptor.

MicroRNA-7 (miR-7) has been reported to target and prevent EGFR production in many instances such as in cancers and during development (Li & Carthew, 2005; Webster *et al*., 2009; Chou *et al*., 2010). *In silico* analysis revealed one highly conserved and two poorly conserved seed sites for miR-7 within the 3′. UTR of EGFR mRNA (Fig. 3A). In order to determine whether or not miR-7 was upregulated in aged fibroblasts, miR-RT followed by QPCR was used. Results showed that miR-7 was found to have a higher expression in aged fibroblasts and in cells stimulated with TGF-β1 when compared to young untreated control cells (Fig. 3B). These data coincide with the downregulation of EGFR mRNA and protein in aged fibroblasts as seen in Fig. 1.

### Overexpression of miR-7 in young fibroblasts causes a loss of EGFR similar to that in aged fibroblasts

**Figure 4:**
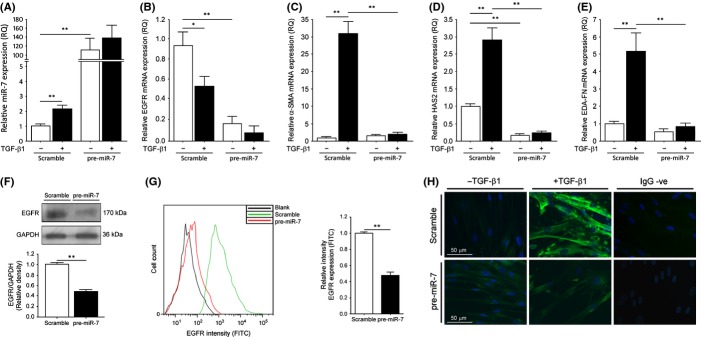
Overexpression of miR-7 in young fibroblasts downregulates EGFR and inhibits differentiation. Young fibroblasts were grown to confluent monolayers and were growth arrested for 24 h before transient transfection with either scrambled pre-miR or pre-miR-7. The relative expressions of (A) miR-7, (B) EGFR mRNA, (C) α-SMA mRNA, (D) HAS2 mRNA, and (E) EDA-FN mRNA were examined by QPCR following incubation in serum-free medium alone or in medium containing 10 ng/mL TGF-β1 for 72 h. Results shown are mean ± SEM of three individual experiments. (F) Western blot analysis of total EGFR protein in young and aged fibroblasts. GAPDH was used as a loading control. Representative blot is shown and densitometry results shown are the mean ± SEM of three individual experiments. (G) Flow cytometry analysis of cell surface expression of EGFR in young (green) and aged (red) fibroblasts. Unlabelled cells (black) were used as intensity controls. Bar graph of relative intensity is shown as the mean ± SEM of three individual experiments. (H) Cells were incubated in serum-free medium alone or in medium containing 10 ng/mL TGF-β1 for 72 h and then analysed by immunocytochemistry for α-SMA. Representative images are shown for three individual experiments. *P ≤ 0.05, **P ≤ 0.01. EGFR, epidermal growth factor receptor. EGFR, epidermal growth factor receptor; α-SMA, α-smooth muscle actin.

Analysis of the effects of overexpression of miR-7 was examined through transfection of pre-miR-7 into young fibroblasts. The relative expression of miR-7 was determined by QPCR and in cells transfected with pre-miR-7 the results showed a significantly large increase in the levels of miR-7 present (Fig. 4A). EGFR mRNA was found to be significantly downregulated in cells transfected with pre-miR-7 (Fig. 4B), while α-SMA (Fig. 4C) and HAS2 (Fig. 4D) mRNA failed to be induced by TGF-β1 treatment. The extra-domain A containing variants of fibronectin (EDA-FN) are associated with differentiation, and its expression is necessary for successful myofibroblast generation (Kohan *et al*., 2010); transfection of pre-miR-7 also prevented the upregulation of EDA-FN mRNA by TGF-β1 (Fig. 4E). These data indicated that miR-7 upregulation had the potential to negatively regulate the essential components involved in fibroblast to myofibroblast differentiation, including those previously described (Simpson *et al*., 2009, 2010; Webber *et al*., 2009; Meran *et al*., 2011). Similarly, both total EGFR protein (Fig. 4F) and EGFR cell surface expression (Fig. 4G) were found to be approximately halved in the presence of pre-miR-7 transfections. To determine whether or not pre-miR-7 transfected cells were still able to differentiate following TGF-β1 stimulation, we used immunocytochemistry for α-SMA. α-SMA fibril formation was lost in cells transfected with pre-miR-7, indicating a prevention of differentiation (Fig 4H).

### Inhibition of miR-7 in aged fibroblasts rescues the TGF-β1-stimulated differentiation response

**Figure 5:**
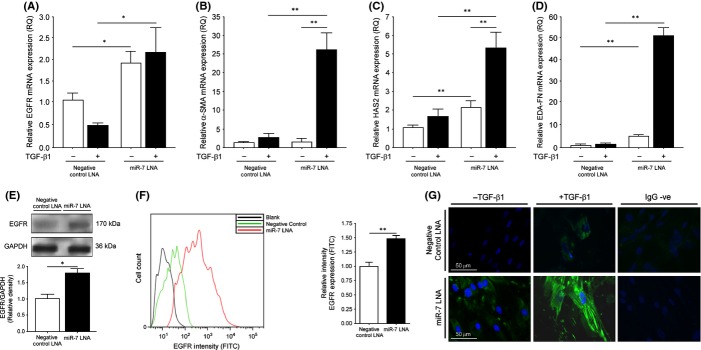
Inhibition of miR-7 with LNA rescues age-associated loss of differentiation. Aged fibroblasts were grown to confluent monolayers and were growth arrested for 24 h before transient transfection with either negative control LNA or miR-7 LNA. The relative expressions of (A) EGFR mRNA, (B) α-SMA mRNA, (C) HAS2 mRNA, and (D) EDA-FN mRNA were examined by QPCR following incubation in serum-free medium alone or in medium containing 10 ng/mL TGF-β1 for 72 h. Results shown are the mean ± SEM of three individual experiments. (E). Western blot analysis of total EGFR protein in young and aged fibroblasts. GAPDH was used as a loading control. Representative blot is shown and densitometry graph shown is mean ± SEM of three individual experiments. (F) Flow cytometry analysis of cell surface expression of EGFR in young (green) and aged (red) fibroblasts. Unlabelled cells (black) were used as intensity controls. Bar graph of relative intensity shown is mean ± SEM of three individual experiments. (G) Cells were incubated in serum-free medium alone or in medium containing 10 ng/mL TGF-β1 for 72 h and then analysed by immunocytochemistry for α-SMA. Representative images are shown for three individual experiments. *P ≤ 0.05, **P ≤ 0.01. EGFR, epidermal growth factor receptor. EGFR, epidermal growth factor receptor; α-SMA, α-smooth muscle actin; LNA, locked nucleic acids.

To test our hypothesis that miR-7 was effectively inhibiting the differentiation response in aged fibroblasts, we transfected miR-7 locked nucleic acids (LNA) to bind and inhibit free miR-7 within our cells. As predicted, EGFR mRNA was upregulated in miR-7 LNA transfected cells compared with negative control LNA transfected cells and did not fall when the cells were treated with TGF-β1 (Fig. 5A). The contributors to myofibroblast differentiation; α-SMA (Fig. 5B), HAS2 (Fig. 5C), and EDA-FN (Fig. 5D) mRNA, were significantly upregulated following TGF-β1 stimulation. Total EGFR protein (Fig. 5E) and cell surface expression (Fig. 5F) were increased in aged fibroblasts transfected with miR-7 LNA. Determination of cellular differentiation was tested, and in cells that had been transfected with miR-7 LNA, there was fluorescence for α-SMA intracellular fibrils (Fig. 5G), indicating that the aged fibroblasts had the ability to differentiate restored if miR-7 was prevented from acting. These data demonstrate the effectiveness of targeting miR-7 in restoring the differentiation response in aged fibroblasts, revealing a potentially valuable mechanism to target in chronic nonhealing wounds in the elderly.

### Inhibition of miR-7 can restore CD44 membrane motility through a HAS2-dependent mechanism

**Figure 6:**
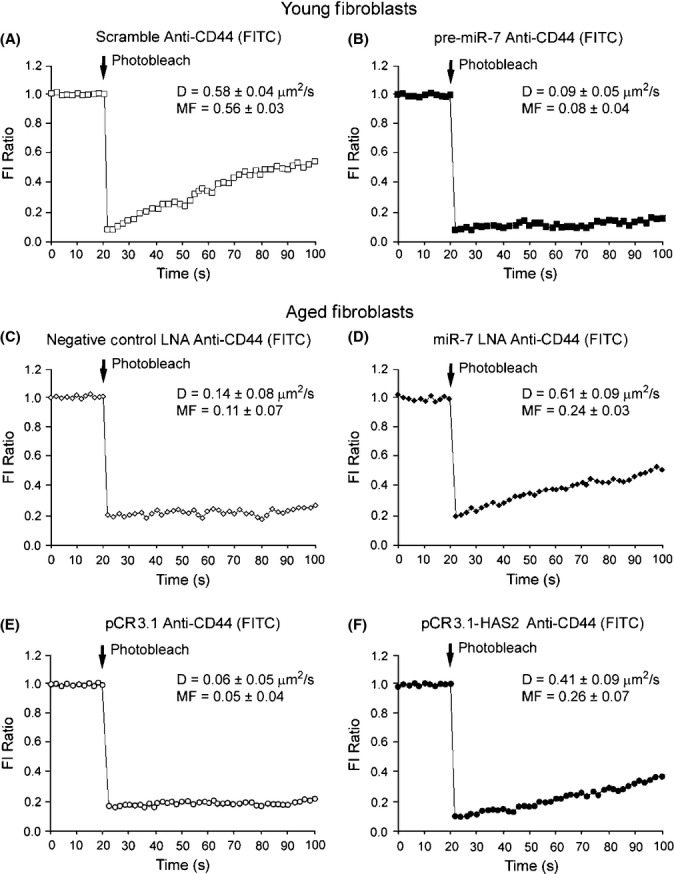
Young fibroblasts overexpressing miR-7 lose CD44 motility, aged fibroblasts transfected with miR-7 inhibitor restore CD44 motility in a similar manner to HAS2 overexpression. Young or aged fibroblasts were grown to 70% confluent monolayers on 22-mm-diameter glass coverslips in 35-mm six-well tissue culture plates. Cells were growth arrested in serum-free medium for 48 h. FRAP was performed at 37 °C by photobleaching an approximately 10 μm area of the cell membrane. The recovery of fluorescence into this area was quantified and expressed as a fraction of the fluorescence intensity (FI) of a second region of membrane outside of the photobleached area (FI Ratio). Complete quantified time-courses, average diffusion constants (D) and mobile fractions (MF) are shown for CD44 in: young cells transfected with (A) scrambled pre-miR or (B) pre-miR-7, and aged cells transfected with (C) negative control LNA, (D) miR-7 LNA, (E) empty pCR3.1 vector, or (F) pCR3.1-HAS2 overexpression vector. All results shown are mean ± SEM of five independent experiments. LNA, locked nucleic acids.

It has been suggested that the loss of CD44 motility in the membrane of aged fibroblasts could prevent the HA-dependent CD44/EGFR interaction necessary for differentiation (Simpson *et al*., 2010; Meran & Steadman, 2011; Meran *et al*., 2011). Observations from the results presented in Fig. 2 showed a loss of CD44 motility in aged fibroblasts; we therefore examined whether overexpression or inhibition of miR-7 would affect CD44 membrane movement. Young fibroblasts were transfected with either a scrambled pre-miR sequence (Fig. 6A) or with pre-miR-7 (Fig. 6B). Interestingly, in cells transfected with pre-miR-7 the potential for CD44 movement throughout the membrane, as highlighted by the diffusion constant (D) and mobile fraction (MF), dropped significantly (both *P* < 0.01) when compared to the scrambled control transfection. These data are indicative of miR-7 having an indirect action on CD44 receptor behaviour. We then examined the inhibition of miR-7 in aged fibroblasts using negative control LNA (Fig. 6C) and miR-7 LNA (Fig. 6D). When aged cells were transfected with miR-7 LNA, the diffusion constant was increased when compared to negative control LNA transfected cells (*P* ≤ 0.05). The MF of CD44, although significantly increased (*P* ≤ 0.05), was not restored to the levels shown in younger fibroblasts (*P* ≤ 0.01 vs. Fig. 2D). We hypothesized that the reason for the restoration seen in miR-7 inhibitory experiments was due to an upregulated the presence of HAS2-produced HA. To test this hypothesis, we transfected aged fibroblasts with an empty vector (Fig. 6E) or with a HAS2 overexpression vector (Fig. 6F). In cells overexpressing HAS2, there was an observable restoration in CD44 diffusion compared with the empty vector control (*P* ≤ 0.05). The MF was also increased (*P* ≤ 0.01), but again not to the full extent of young fibroblasts (*P* ≤ 0.05 vs. Fig. 2D). These data illustrate an alternative mechanism through which miR-7 can regulate the differentiation response. The inhibition of HAS2 mRNA when overexpressing miR-7 in young fibroblasts (Fig. 4D) and the restoration of HAS2 mRNA expression in aged fibroblasts transfected with miR-7 LNA (Fig. 5C) suggest that there may be a HA-dependent regulation of CD44 membrane motility that is operational in young but not aged fibroblast cells.

## Discussion

This report provides additional mechanisms and insights into the age-associated loss of TGF-β1- dependent fibroblast to myofibroblast differentiation. The importance of EGFR and its loss in cellular aging has been described recently and been shown to have a significant effect on the myofibroblast differentiation pathway (Meran *et al*., 2007; Simpson *et al*., 2010; Meran & Steadman, 2011), going some way towards explaining the mechanisms of resistance to TGF-β1-stimulated differentiation (Shiraha *et al*., 2000; Simpson *et al*., 2010). In light of these reports, we sought to determine the extent of the loss of EGFR in our aged fibroblast cells and further detail the EGFR deficiency at multiple levels including its transcriptional, translational, and cell surface expression. Figure S1 further highlighted the importance of functional EGFR signalling in the differentiation response. The data reported here support previous findings of the age-associated loss of EGFR at the mRNA and protein levels. However, the activity of the EGFR promoter was not found to be significantly different between young and aged fibroblasts. The maintenance of EGFR promoter activity indicated that the transcription factors involved in EGFR transcription were unlikely to be affected in an age- dependent manner and that there was another factor responsible for the loss of EGFR mRNA, protein, and cell-surface expression. We therefore hypothesized that age-associated loss of EGFR could be regulated through the presence of mRNA inhibitors such as miRNAs.

The results in this study also illustrated an age-associated loss of CD44 receptor membrane motility as demonstrated by FRAP confocal microscopy. The HA receptor CD44 can function as a co- receptor, physically associating with several membrane-bound proteins, resulting in modulation of intracellular signal transduction pathways and facilitating the formation of specialized signalling complexes (Wang & Bourguignon, 2006a,b; Palyi-Krekk *et al*., 2008). The ability to do so with EGFR plays a key role in myofibroblast differentiation (Midgley *et al*., 2013). While CD44 was free to diffuse within the plasma membrane of young fibroblasts, the ability of CD44 to diffuse freely was attenuated in aged cells. As the loss of HA production by aged cells has also been described previously (Simpson *et al*., 2009), we determined CD44 mobility to be HA- dependent, and thus, a loss of overall HA could contribute to the loss of CD44 cell membrane movement in aged fibroblasts. Figure S2 demonstrated that removal of HA production resulted in a loss of CD44 membrane motility.

Multiple reports have described miR-7 as having an important role in the regulation of EGFR in cancer and development (Li & Carthew, 2005; Kefas *et al*., 2008; Chou *et al*., 2010). However, this relationship between miR-7 and EGFR has not been described in other areas of EGFR function, such as fibroblast differentiation or age-associated loss of phenotype. *In silico* analysis identified the highly reflect the downregulated EGFR mRNA present in aged fibroblasts. Additionally, an interesting observation was made in the young fibroblast group, wherein miR-7 was upregulated and EGFR mRNA downregulated in response to TGF-β1. Whether this change to miR-7 and in turn EGFR mRNA is directly modulated by TGF-β1 stimulation is not currently known and requires further investigation; however, a possible mechanism of miR-7 activity has been shown through its promotion by EGFR signalling and c-Myc (Chou *et al*., 2010).

Interestingly, through miR-7 overexpression in young fibroblasts, we were able to mimic the decreased expression of EGFR observed in aged fibroblasts, further supporting a role for miR-7 in age-associated regulation of EGFR and the differentiation response. Furthermore, the inhibition of miR-7 in aged fibroblasts significantly induced EGFR mRNA, protein, and cell-surface expression, together with HAS2 mRNA. Additionally, in response to TGF-β1 treatment in the presence of miR-7 inhibition, there was successful upregulation of α-SMA fibril formation and induction of α-SMA, HAS2, and EDA-FN mRNA, indicating restored differentiation of fibroblasts to myofibroblasts. The importance of HA-CD44 in mediating differentiation (Sherman *et al*., 1994; Bourguignon *et al*., 2006) and the TGF-β1-induced myofibroblast response (Meran *et al*., 2007; Simpson *et al*., 2009, 2010; Webber *et al*., 2009; Meran & Steadman, 2011) has been previously documented. We were interested in examining CD44 in the cellular membrane of young and aged cells in response to miR-7 overexpression and inhibition, respectively. Overexpression of miR-7 in young cells resulted in diffusion rates of CD44 declining to similar levels as those found in aged fibroblasts. In addition, the inhibition of miR-7 in aged fibroblasts restored the diffusion rates of CD44 to levels observable in young fibroblasts. As inhibition of miR-7 with LNA was able to upregulate both EGFR and HAS2 mRNA, we tested the hypothesis that overexpression of either in aged fibroblasts could restore CD44 movement. Although overexpression of EGFR had no effect on CD44 motility (data not shown), HAS2 overexpression did restore CD44 movement to rates observable in young fibroblasts and aged fibroblasts transfected with miR-7 LNA. Therefore, we propose that HAS2 synthesis of HA is an important mechanism-mediating CD44 lateral movement throughout the cellular membrane and that miR-7 can regulate HAS2 expression through the inhibition of EGFR and components of its signalling pathway, therefore indirectly affecting CD44 movement in order to prevent cellular differentiation. Indeed, miR-7 has been shown to inhibit the Akt/mTOR pathway downstream of EGFR signalling (Kefas *et al*., 2008; Webster *et al*., 2009), and an association between Akt and HAS2 production has previously been illustrated (Kultti *et al*., 2010; Bernert *et al*., 2011).

Explanation of how miR-7 inhibition could rescue age-associated loss of differentiation while EGFR overexpression alone could not (Simpson *et al*., 2010) may be a result of the mRNA targets of miR-7. *In silico* analysis of miR-7 seed sites revealed potential targets on neither HAS2, α-SMA nor CD44. However, there were several targets of miR-7 within the EGFR-signalling pathway, the activation of these are necessary for HAS2 induction, the reorganization of the actin cytoskeleton, α- SMA upregulation and myofibroblast differentiation. These targets included MAPK/ERK, CaMKII, Rho-GTPase, PI3K, Akt and mTOR. EGFR overexpression in aged fibroblasts (Simpson *et al*., 2010) failed to successfully restore differentiation; it is possible that this was due to an elevated expression of miR-7 in aged cells, not only inhibiting EGFR mRNA but also the production of its downstream targets and thus HAS2 and α-SMA gene induction. Bypassing the EGFR pathway and also overexpressing HAS2 could restore differentiation, and this is likely through the increased production of HA, restoration of CD44 membrane motility and its interaction with EGFR, solidifying the signalling response of EGFR and providing sufficient activity to trigger the differentiation response in aged fibroblasts.

We propose a mechanism through which miR-7 upregulation in aged cells is able to downregulate EGFR protein and expression through targeting EGFR mRNA for breakdown and has additional targets within the EGFR-dependent signalling pathway that directly regulate HAS2 production. The consequence of miR-7 activity therefore results in the downregulation of the key cellular components; EGFR and HAS2, involved in fibroblast to myofibroblast differentiation. Additionally, as a direct result of reduced HAS2 expression, there is a reduction in HA production and binding to CD44, compromising its ability to move throughout the cellular membrane and thus a reduction in its interaction with EGFR in response to TGF-β1 (Wang & Bourguignon, 2006b; Simpson *et al*., 2010; Meran *et al*., 2011), an essential step in driving differentiation.

The loss of cellular phenotype and response to stimulation is a pivotal change in cellular aging. Age-related loss of fibroblast-to-myofibroblast differentiation is often associated with failure of normal wound healing and contraction leading to chronic nonhealing wounds in the elderly. Chronic nonhealing wounds have been estimated to affect 4% of the UK population over the age of 65, and the morbidity associated with this impaired wound healing is estimated to cost the health service in excess of £1 billion annually in the United Kingdom (Ashcroft *et al*., 2002) and $9 billion in the United States (Ashcroft & Roberts, 2000). With the costs rising in correlation with the aging population’s ever-increasing life spans; chronic wounds pose a real problem to present and future healthcare across the world. In this study, we report a novel insight into the targeting of miR-7 in aged fibroblasts, to rescue and promote differentiation into myofibroblasts and therefore drive effective wound healing in the elderly.

## Experimental procedures

### Materials

All reagents were from Sigma-Aldrich (Poole, UK) unless otherwise stated. The primary antibodies and dilutions used for western blot analysis, immunocytochemistry, flow cytometry and confocal microscopy were monoclonal mouse anti-EGFR (1:1000) from Calbiochem (Darmstadt, Germany), monoclonal mouse anti-α-SMA (1:50) from DAKO (Aachen, Germany), monoclonal mouse anti-EGFR-FITC (1:1000) and monoclonal rat anti-CD44-FITC (1:1000) from Abcam (Cambridge, UK), respectively. Reverse transcription, pre-miR-7 and pre-miR-Scramble transfection reagents, and QPCR primers and reagents were purchased from Invitrogen and Applied Biosystems (Cheshire, UK). Other reagents used were recombinant TGF-β1 from R&D Systems (Abingdon, UK), and miR-7 and negative control miR miRCURY LNA from Exiqon (Denmark, EU).

### Cell culture

Primary human lung fibroblasts (AG02262; NIA Aging Cell Respiratory Corriel Institute, Camden, NJ, USA) were cultured in Dulbecco’s modified Eagle’s medium (DMEM) and F-12 medium containing 2 mm l-glutamine, 100 units/mL penicillin, and 100 μg/mL streptomycin supplemented with 10% foetal calf serum (FCS; Biologic Industries Ltd., Cumbernauld, UK). The cells were maintained at 37 °C in a humidified incubator in an atmosphere of 5% CO_2_, and fresh growth medium was added to the cells every 3–4 days until confluence. Cells were growth arrested in serum-free medium for 48 h before use in experiments, and all experiments were performed under serum-free conditions unless otherwise stated. All experiments involving young fibroblasts used cells at passages 6–8 [population doubling level (PDL) 15–20]; while those involving aged fibroblasts used cells at presenescent late passages 14–15 (PDL 25–28); cells underwent senescence at PDL 30 as determined by growth curves (data not shown). All experiments were performed on confluent cultures except for those experiments using antibody visualization, subconfluent cells of approximately 70% confluence were used for optimal antibody coverage. Myofibroblasts were differentiated by the incubation of fibroblast cultures in serum-free medium containing 10 ng/mL TGF-β1 for 72 h.

### Reverse transcription (RT) and real-time quantitative polymerase chain reaction (RT-QPCR)

RT and QPCR-RT were used to assess EGFR, α-SMA, HAS2 and EDA-FN mRNA expression in fibroblasts or myofibroblasts. The cells were grown in 35-mm dishes and washed with PBS prior to transcription was performed using the high-capacity cDNA reverse transcription kits according to the manufacturer’s protocol (Applied Biosystems, Cheshire, UK). This uses the random primer method for initiating cDNA synthesis. As a negative control, RT was performed with sterile H_2_O replacing the RNA sample. QPCR was performed as in our previous studies (Simpson *et al*., 2010) using the 7900HT Fast Real-Time PCR System (Applied Biosystems). Ribosomal RNA (rRNA) was used as an endogenous control. MicroRNA-RT and QPCR were performed for miR-7 according to TaqMan MicroRNA Assays (Applied Biosystems) and miR-16 was used as an endogenous control.

### Western blot analysis

Western blot analysis was used to assess expression of total EGFR. Cells were grown to confluence in 35-mm dishes; total protein was extracted in RIPA lysis buffer containing 1% PIC, 1% PMSF and 1% SO (Santa Cruz Biotechnology, CA, USA). Western blot analysis was carried out as described in our previous work (Simpson *et al*., 2010). The nitrocellulose membranes were incubated with the appropriate primary antibodies in 1% BSA, 0.1% Tween-PBS. Expression of GAPDH was analysed as a control to ensure equal loading (rabbit anti-GAPDH; 1:5000 dilution). The secondary antibodies used were anti-mouse IgG/horseradish peroxidize conjugate (Abcam, Cambridge, UK; 1:5000 dilution in 1% BSA, 0.1% Tween-PBS), and anti-rabbit IgG/horseradish peroxidize conjugate (Cell Signalling Technology, Beverly, MA; 1:5000 dilution in 1% BSA, 0.1% Tween-PBS). Detection was performed using ECL reagent (GE Healthcare, Amersham, UK).

### Flow cytometry

Young and aged fibroblasts were grown to confluence and growth arrested in serum-free medium for 48 h. Cells were washed with PBS and incubated with 0.01% trypsin-EDTA to lift the cells. Trypsin was subsequently neutralized with FCS, and the cell solution was centrifuged at 1500 *g* for 5 min at 20 °C. The supernatant was aspirated and the cell pellet resuspended in 1% BSA-PBS containing the primary fluorophore-conjugated antibody for 30 min on ice, before washes and resuspension in 1% BSA-PBS. Flow cytometry was performed using a FACSCanto II flow cytometer (BD Biosciences), and data were analysed using FlowJo version 7 (Tree Star, Ashland, OR, USA).

### Luciferase reporter plasmid generation

A 200 bp insert encoding the EGFR promoter was PCR amplified using the Phusion DNA polymerase system (New England Biolabs, Herts, UK) from the forward primer; 5′-ACTCCCGCCGGAGACTAGGT-3′ and the reverse primer; 5′- CCGCGTCGGGCGCTCACACC-3′ with the addition of KpnI and XhoI endonuclease restriction sites, respectively. Amplified promoter inserts were PCR purified, digested with the appropriate endonuclease restriction enzymes (New England Biolabs) for 2 h at 37 °C, electrophoresed and extracted from 1% agarose gel according to Qiagen gel extraction kit protocol (Qiagen, Barcelona, Spain). Inserts were then ligated together with KpnI and XhoI digested pGL3Basic (pGL3b) vector using the T4 DNA ligase system (New England Biolabs) overnight at 16 °C. The pGL3b plasmid containing the 200 bp EGFR promoter insert (pGL3b-EGFR) was then transformed using the 42 °C heat-shock method into one-shot competent *Escherichia coli* (New England Biolabs) and grown overnight on ampicillin containing agar. Single colonies were extracted, cloned and DNA purified according to the Miniprep Kit protocol (Sigma-Aldrich). Cloned pGL3b-EGFR vector uptake of insert was confirmed with DNA sequencing.

### HAS2-overexpression vector generation

The HAS2 open-reading frame (Simpson *et al*., 2009) was inserted into the vector pCR3.1 using a standard ligation reaction with T4 DNA ligase (New England Biolabs). Amplification of the cloned vector was performed via bacterial transformation into one-shot competent *Escherichia coli* (New England Biolabs) and grown overnight on ampicillin containing agar. Single colonies were extracted, cloned and DNA purified according to the Miniprep Kit protocol (Sigma-Aldrich). Cloned pCR3.1-HAS2 vector uptake of insert was confirmed with DNA sequencing. Negative RT experiments were performed alongside HAS2 mRNA QPCR to ensure that the pCR-3.1-HAS2 was not conveying false-positive overexpression.

### Transfection

Plasmid pGL3b-EGFR, pCR3.1-HAS2 and miRCURY LNA transfections were completed using the Lipofectamine LTX system (Invitrogen, Paisley, UK) according to protocol following optimization of transfection. Pre-miR-7 transfections were completed using the Lipofectamine 2000 system (Invitrogen) according to the protocol following optimization. Transfection optimization and efficiency was determined by co-transfection of GFPmax vector (Amaxa, Cologne, Germany). Cells were incubated for 24 h following transfection prior to continuation of experiments.

### Luciferase reporter analysis

Reporter analysis was performed 24 h after transfection using the Dual-Luciferase reporter assay kit (Promega, Southampton, UK) and detected with a FLUOstar OPTIMA plate reader (BMG Labtech, UK). Renilla luciferase was co-transfected with pGL3b-EGFR and used as a control for transfection efficiency and for normalization of data.

### Immunocytochemistry

Cells were grown to 70% confluence in eight-well glass chamber slides. The culture medium was removed, and the cells were washed with sterile PBS prior to fixation in 4% paraformaldehyde for 10 min at room temperature. To ensure visualization of cytoskeletal proteins, fixed slides were permeabilized with 0.1% Triton X-100 in PBS for 10 min when necessary. Slides were washed with PBS and then blocked with 1% BSA for 30 min prior to a further washing step. Subsequently, the slides were incubated with the primary antibody diluted in 0.1% BSA/PBS overnight at 4 °C. Following a further wash step, slides were incubated with secondary antibodies for 1 h at room temperature in darkness (anti-mouse IgG/AlexaFluor 488; Invitrogen). Cells were then mounted and analysed by fluorescent microscopy.

### Laser confocal microscopy and fluorescence recovery after photobleaching

Cells were grown to 70% confluence on 22-mm-diameter glass coverslips in 35-mm dishes. Following appropriate experimental methods, the coverslip was removed from the medium and mounted on a heated microscope stage at 37 °C. Five hundred microlitres of medium was placed onto the mounted coverslip and the primary fluorophore-conjugated antibody was added. Analysis was performed by laser confocal microscopy and FRAP.

Leica Confocal Software (Leica Microsystems, Hamburg GmbH) was used to assess FRAP data and generate fluorescence intensity ratios (FI ratio); defined as FI ratio = *F*_*z*_/*F*_*o*_, Where *F*_*z*_ is the intensity of the photobleached zone, and *F*_*o*_ is the intensity of the control region taken from outside of the photobleached zone. Average diffusion constants (D) were calculated from the D = (*w*^2^/2*t*_*½*_)γD equation Where *w* is the radius of the photobleached area, *t*_*½*_ is the half time of fluorescent recovery and γD is a constant that is dependent on experimental conditions (Axelrod *et al*., 1976). Mobile fractions (MF) represent the fraction of receptors that had the ability to recover into the photobleached zone over the observed time.

### Statistical analysis

Western blot images were densitometrically analysed by ImageJ (NIH Software, Bethesda, MD, USA). Graphical data are expressed as means ± SEM. The unpaired two-tailed Student’s *t-*test was used to identify statistical significance. Data were analysed using the software MiniTab version 15.0 (Minitab Solutions, State College, PA, USA), and *P ≤ 0.05; **P ≤ 0.01 was considered significant.

## Supporting Information

**Fig S1.** Inhibition of EGFR prevented upregulation of key differentiation components by TGF-β1. **Fig S2.** Inhibition of HA synthesis resulted in loss of CD44 membrane motility.

## References

[b1] Ashcroft GS, Roberts AB (2000). Loss of Smad3 modulates wound healing. Cytokine Growth Factor Rev.

[b2] Ashcroft GS, Horan MA, Ferguson MW (1995). The effects of ageing on cutaneous wound healing in mammals. J. Anat.

[b3] Ashcroft GS, Mills SJ, Ashworth JJ (2002). Ageing and wound healing. Biogerontology.

[b4] Axelrod D, Koppel DE, Schlessinger J, Elson E, Webb WW (1976). Mobility measurement by analysis of fluorescence photobleaching recovery kinetics. Biophys. J.

[b5] Bernert B, Porsch H, Heldin P (2011). Hyaluronan synthase 2 (HAS2) promotes breast cancer cell invasion by suppression of tissue metalloproteinase inhibitor 1 (TIMP-1). J. Biol. Chem.

[b6] Bourguignon LY, Ramez M, Gilad E, Singleton PA, Man MQ, Crumrine DA, Elias PM, Feingold KR (2006). Hyaluronan-CD44 interaction stimulates keratinocyte differentiation, lamellar body formation/secretion, and permeability barrier homeostasis. J. Invest. Dermatol.

[b7] Chou YT, Lin HH, Lien YC, Wang YH, Hong CF, Kao YR, Lin SC, Chang YC, Lin SY, Chen SJ, Chen HC, Yeh SD, Wu CW (2010). EGFR promotes lung tumorigenesis by activating miR-7 through a Ras/ERK/Myc pathway that targets the Ets2 transcriptional repressor ERF. Cancer Res.

[b8] Gabbiani G (2003). The myofibroblast in wound healing and fibrocontractive diseases. J. Pathol.

[b9] Ito T, Williams JD, Fraser D, Phillips AO (2004a). Hyaluronan attenuates transforming growth factor-beta1-mediated signaling in renal proximal tubular epithelial cells. Am. J. Pathol.

[b10] Ito T, Williams JD, Fraser DJ, Phillips AO (2004b). Hyaluronan regulates transforming growth factor-beta1 receptor compartmentalization. J. Biol. Chem.

[b11] Kefas B, Godlewski J, Comeau L, Li Y, Abounader R, Hawkinson M, Lee J, Fine H, Chiocca EA, Lawler S, Purow B (2008). microRNA-7 inhibits the epidermal growth factor receptor and the Akt pathway and is down-regulated in glioblastoma. Cancer Res.

[b12] Kohan M, Muro AF, White ES, Berkman N (2010). EDA-containing cellular fibronectin induces fibroblast differentiation through binding to alpha4beta7 integrin receptor and MAPK/Erk 1/2-dependent signaling. FASEB J.

[b13] Kultti A, Karna R, Rilla K, Nurminen P, Koli E, Makkonen KM, Si J, Tammi MI, Tammi RH (2010). Methyl-beta-cyclodextrin suppresses hyaluronan synthesis by down-regulation of hyaluronan synthase 2 through inhibition of Akt. J. Biol. Chem.

[b14] Li X, Carthew RW (2005). A microRNA mediates EGF receptor signaling and promotes photoreceptor differentiation in the *Drosophila* eye. Cell.

[b15] Meran S, Steadman R (2011). Fibroblasts and myofibroblasts in renal fibrosis. Int. J. Exp. Pathol.

[b16] Meran S, Thomas D, Stephens P, Martin J, Bowen T, Phillips A, Steadman R (2007). Involvement of hyaluronan in regulation of fibroblast phenotype. J. Biol. Chem.

[b17] Meran S, Luo DD, Simpson R, Martin J, Wells A, Steadman R, Phillips AO (2011). Hyaluronan facilitates transforming growth factor-beta1-dependent proliferation via CD44 and epidermal growth factor receptor interaction. J. Biol. Chem.

[b18] Midgley A, Rogers M, Hallett M, Clayton A, Bowen T, Phillips A, Steadman R (2013). TGF-β1-stimulated fibroblast to myofibroblast differentiation is mediated by HA-facilitated EGFR and CD44 co-localisation in lipid rafts. J. Biol. Chem.

[b19] Palyi-Krekk Z, Barok M, Kovacs T, Saya H, Nagano O, Szollosi J, Nagy P (2008). EGFR and ErbB2 are functionally coupled to CD44 and regulate shedding, internalization and motogenic effect of CD44. Cancer Lett.

[b20] Sherman L, Sleeman J, Herrlich P, Ponta H (1994). Hyaluronate receptors: key players in growth, differentiation, migration and tumor progression. Curr. Opin. Cell Biol.

[b21] Shiraha H, Gupta K, Drabik K, Wells A (2000). Aging fibroblasts present reduced epidermal growth factor (EGF) responsiveness due to preferential loss of EGF receptors. J. Biol. Chem.

[b22] Simpson RM, Meran S, Thomas D, Stephens P, Bowen T, Steadman R, Phillips A (2009). Age- related changes in pericellular hyaluronan organization leads to impaired dermal fibroblast to myofibroblast differentiation. Am. J. Pathol.

[b23] Simpson RM, Wells A, Thomas D, Stephens P, Steadman R, Phillips A (2010). Aging fibroblasts resist phenotypic maturation because of impaired hyaluronan-dependent CD44/epidermal growth factor receptor signaling. Am. J. Pathol.

[b24] Wang SJ, Bourguignon LY (2006a). Hyaluronan-CD44 promotes phospholipase C-mediated Ca2 + signaling and cisplatin resistance in head and neck cancer. Arch. Otolaryngol. Head Neck Surg.

[b25] Wang SJ, Bourguignon LY (2006b). Hyaluronan and the interaction between CD44 and epidermal growth factor receptor in oncogenic signaling and chemotherapy resistance in head and neck cancer. Arch. Otolaryngol. Head Neck Surg.

[b26] Webber J, Jenkins RH, Meran S, Phillips A, Steadman R (2009). Modulation of TGFbeta1- dependent myofibroblast differentiation by hyaluronan. Am. J. Pathol.

[b27] Webster RJ, Giles KM, Price KJ, Zhang PM, Mattick JS, Leedman PJ (2009). Regulation of epidermal growth factor receptor signaling in human cancer cells by microRNA-7. J. Biol. Chem.

