# Supplementary material for: MicroRNA-7 inhibition rescues age-associated loss of epidermal growth factor receptor and hyaluronan-dependent differentiation in fibroblasts
Source: Aging Cell. 2013 Nov 12;13(2):235–44. doi: 10.1111/acel.12167 (PMC4331777; doi:10.1111/acel.12167)

**Supplementary Data**

**Figure S1**. *Inhibition of EGFR prevented upregulation of key differentiation components by TGF-*β*1*. Growth arrested fibroblasts were treated with the inhibitor of EGFR phosphorylation, AG1478 (10µM), for 1 hour prior to incubation with serum-free media alone (white bars) or serum-free media containing TGF-β1 (10ng/ml; black bars). When compared to untreated cells, those treated with AG1478 had no upregulation of HAS2 (A), α-SMA (B), or EDA-FN (C) indicating that the induction of these genes requires EGFR phosphorylation activity. ***P=<0.01*.


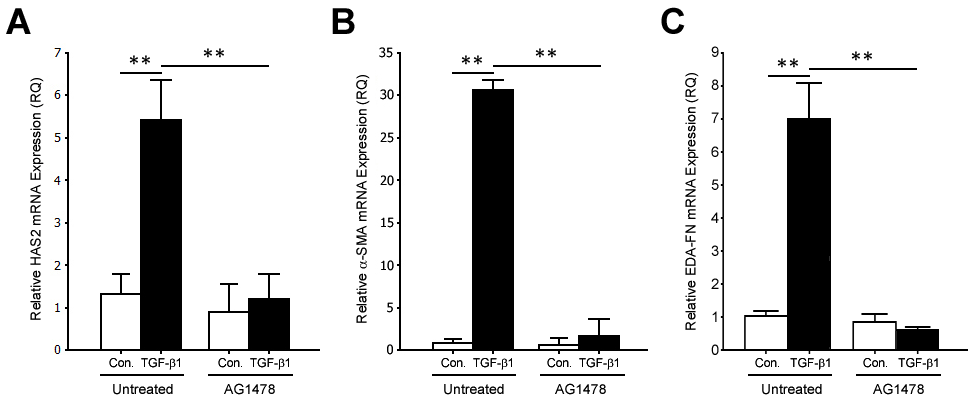


**Figure S2**. *Inhibition of HA synthesis resulted in loss of CD44 membrane motility*. Fibroblasts were growth arrested and treated with serum-free media containing 0.6% DMSO (v/v) as a control or

0.5µM 4-methylumbelliferone (4MU) for 24 hours prior to FRAP analysis of CD44 membrane motility. Control fibroblasts (A) had rapid CD44 diffusion rates and a mobile fraction of approximately 60%, whilst cells treated with 4MU (B) had significantly reduced diffusion rates (P=<0.01) and a mobile fraction of approximately 8% (P=<0.01 vs. A). Data illustrates that cellular production of HA is required for CD44 to have the potential of membrane motility.


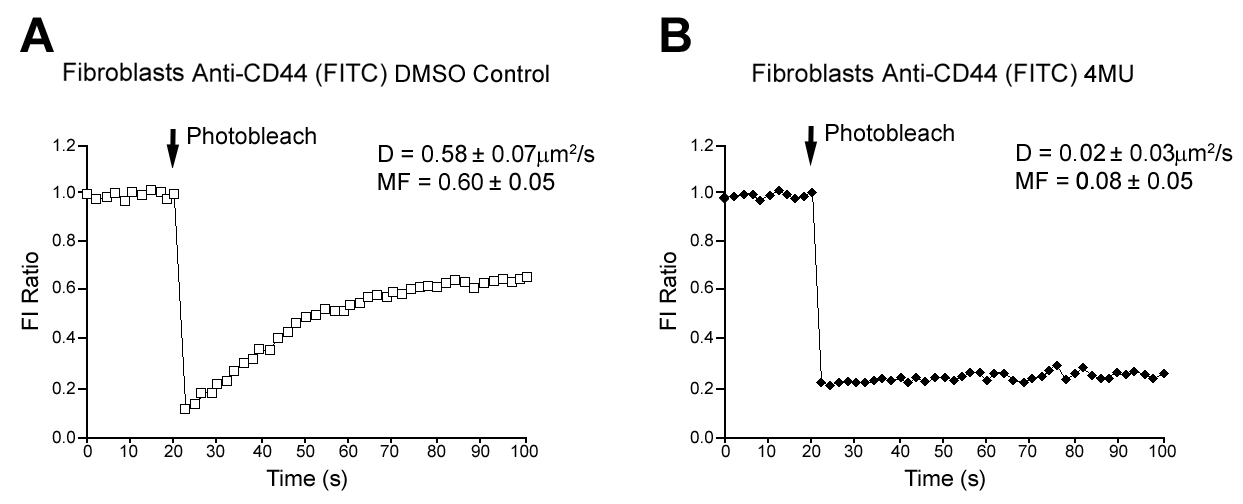

Supplement: Supplementary file 1 — Fig S1. Inhibition of EGFR prevented upregulation of key differentiation components by TGF-β1. Fig S2. Inhibition of HA synthesis resulted in loss of CD44 membrane motility. [file acel0013-0235-sd1.docx]
